# A feature selection-based framework to identify biomarkers for cancer diagnosis: A focus on lung adenocarcinoma

**DOI:** 10.1371/journal.pone.0269126

**Published:** 2022-09-06

**Authors:** Omar Abdelwahab, Nourelislam Awad, Menattallah Elserafy, Eman Badr

**Affiliations:** 1 University of Science and Technology, Zewail City of Science and Technology, Giza, Egypt; 2 Center of Informatics Science, Nile university, Giza, Egypt; 3 Center for Genomics, Helmy Institute for Medical Sciences, Zewail City of Science and Technology, Giza, Egypt; 4 Faculty of Computers and Artificial Intelligence, Cairo University, Giza, Egypt; Chinese Academy of Sciences, CHINA

## Abstract

Lung cancer (LC) represents most of the cancer incidences in the world. There are many types of LC, but Lung Adenocarcinoma (LUAD) is the most common type. Although RNA-seq and microarray data provide a vast amount of gene expression data, most of the genes are insignificant to clinical diagnosis. Feature selection (FS) techniques overcome the high dimensionality and sparsity issues of the large-scale data. We propose a framework that applies an ensemble of feature selection techniques to identify genes highly correlated to LUAD. Utilizing LUAD RNA-seq data from the Cancer Genome Atlas (TCGA), we employed mutual information (MI) and recursive feature elimination (RFE) feature selection techniques along with support vector machine (SVM) classification model. We have also utilized Random Forest (RF) as an embedded FS technique. The results were integrated and candidate biomarker genes across all techniques were identified. The proposed framework has identified 12 potential biomarkers that are highly correlated with different LC types, especially LUAD. A predictive model has been trained utilizing the identified biomarker expression profiling and performance of 97.99% was achieved. In addition, upon performing differential gene expression analysis, we could find that all 12 genes were significantly differentially expressed between normal and LUAD tissues, and strongly correlated with LUAD according to previous reports. We here propose that using multiple feature selection methods effectively reduces the number of identified biomarkers and directly affects their biological relevance.

## Introduction

Detecting the most correlated genes to a specific disease has been a major computational problem. Standard statistical methods such as t-test, linear regression, or negative binomial distribution are used to identify differentially expressed genes, providing a large number of candidate genes [1–3]. However, only a few of these candidates contribute significantly to the pathology and response to treatment. Therefore, feature selection (FS) techniques have been utilized to identify potential gene biomarkers whose expression profiling can help in phenotypic differentiation [4–8]. FS techniques are used to identify genes whose transcriptomic profiling varies significantly across sample groups. Feature selection reduces the dimensionality of the input data before constructing a predictive model without losing relevant information. Additionally, it increases the speed of learning, facilitates generalization, and improves performance [9]. Utilizing feature selection with large scale data such as RNA-seq allows important feature extraction and overcomes the “curse of dimensionality” problem. The curse of dimensionality appears when the number of data features increases, along with much smaller data size, as in the RNA-seq data case. Although a higher number of features should allow more information, practically, it includes more redundant and possibly noisy data. More complex models are required to handle such high dimension data, which can lead to overfitting [10–12]. Thus, employing multiple feature selection techniques effectively decreases the number of utilized features and identifies the most significant ones.

Different studies have utilized feature selection to detect the transcriptomic signature of different diseases. Huijuan et al. introduced a hybrid FS technique that combines both mutual information maximization and adaptive genetic algorithm. DNA microarray data of six cancer sets have been analyzed. The authors showed that utilizing multiple techniques increased classification accuracy and reduced feature dimensionality [4]. Tabl et al. used Chi-square and Info-Gain along with a tree-based model to predict the 5-year survivability of breast cancer patients [11]. Li et al. utilized the mutual information method and then the incremental feature selection along with a support vector machine (SVM) classifier and selected 23 discriminative genes for Osteoarthritis, where 97.1% accuracy was achieved [13]. Chen et al. utilized the Monte-Carlo feature selection method with SVM classifier to identify gene expression signatures in multiple types of neural stem cells [14] (the hybrid feature selection methods are reviewed in [11]).

Developing a reliable computational approach to determine gene expression signature improves the diagnosis of complex diseases, as a small number of correlated genes can be exploited and further investigated in clinical settings. This is especially important for developing countries, where RNA-seq and transcriptome profiling of patients’ samples are not affordable to decide on the best therapeutic approach. Thus, analyzing a small set of candidate genes will contribute to more accurate therapy prescription, in a cost-efficient manner.

In this article, we are proposing a framework where a combination of feature selection methods and a prediction model are utilized to detect biomarker profiling that differentiates between normal and lung adenocarcinoma cancer patients. We selected Lung cancer (LC) as it is one of the most prevalent malignancies worldwide and the most common cause of global cancer-associated mortality, with a five-year survival rate. Lung adenocarcinoma (LUAD) is a subtype of lung cancer whose causes are still ambiguous. One of the possible causes might be deficiencies in therapeutic methods and difficulties in early diagnosis. The early diagnosis of cancer contributes to increasing the survival rate, which makes it important to create other diagnostic tools for LUAD [15].

In an attempt to identify the most significantly correlated genes to LUAD, we utilized mutual information (MI) [16] and recursive feature elimination (RFE) feature selection techniques along with the SVM classification model [17]. In addition, we have also utilized Random Forest (RF) as an embedded FS technique [17].

Our framework takes advantage of filter, wrapper, and embedded feature selection methods. As filter techniques focus mainly on the statistical characteristics of the input data, the features are selected based on the correlation between the feature and the target class independent of a classification model. MI was utilized to measure the relevance of the features to the classes and the redundancy among them, which reduces the number of highly correlated features. However, it produces a relatively large number of features. Utilizing a wrapper-based technique where MI was employed with SVM as a classification model significantly reduced the selected features. In this case, the features are selected based on the SVM performance. RFE is another well-known feature reduction technique widely used in machine learning to reduce high dimensional data despite its high computational time [17–22]. Finally, Random Forest (RF) is used as an embedded technique where feature selection is a part of the classifier construction process. RF is not sensitive to outliers, it reduces feature correlations, but it is prone to overfitting [23, 24]. All previous methods have been utilized to identify a specific subset of features as candidate biomarkers. Utilizing multiple FS techniques maximizes their advantages and alleviates their disadvantages. We hypothesize that consensus features among all FS methods yield the most significant biomarkers.

Interestingly, we could observe noticeable variations in each technique’s candidate genes but identifying the common candidates between all techniques yielded 12 genes that are strongly correlated with LUAD, as illustrated later in the discussion section. DEseq2 [25] has been utilized for results verification. It is a standard pipeline that is very commonly used by biologists. Its results are reliable and robust to outliers [26, 27]. Upon performing differential gene expression analysis using DEseq2, the 12 genes were found to be significantly differentially expressed between LUAD and normal samples. Our predictive model trained on gene biomarker profiling achieves an accuracy of 97.99% and is capable of identifying candidates that are highly correlated to LUAD.

## Results

### A framework to identify genes highly correlated to LUAD

**Fig 1:**
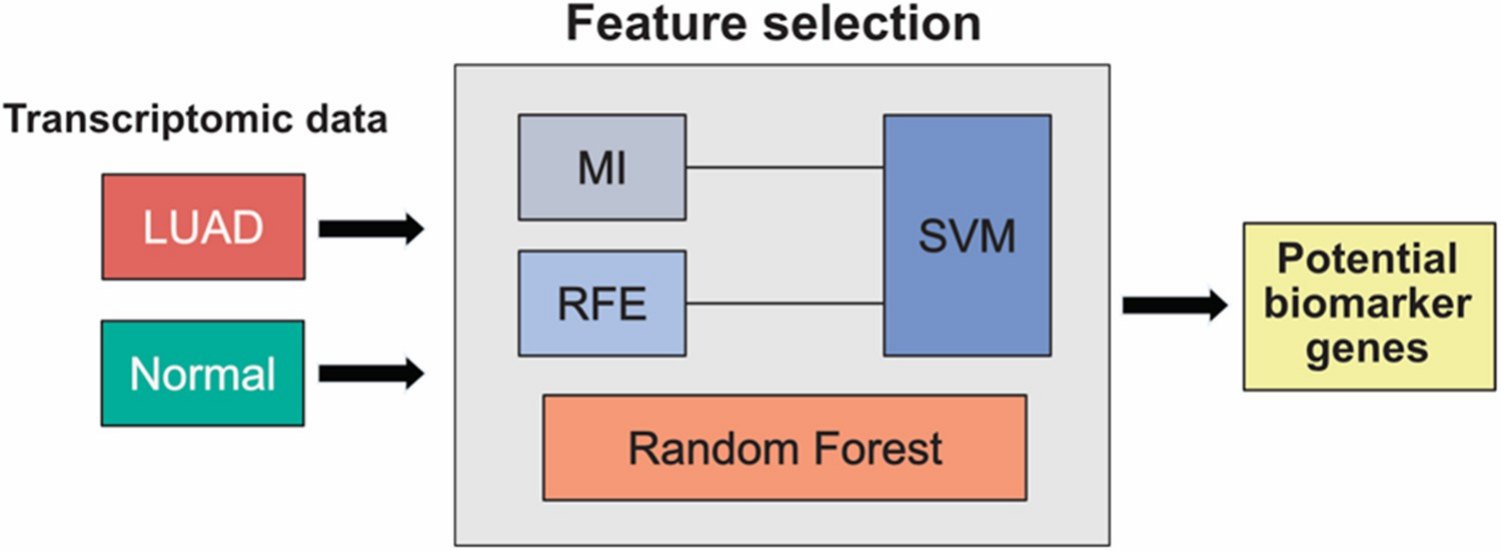
An overview of our proposed framework.

In this study, we propose a framework that applies three feature selection techniques to identify genes highly correlated to LUAD (Fig 1). The LUAD RNA-seq data was obtained from The Cancer Genome Atlas (TCGA-LUAD). Each technique was utilized separately along with SVM classification model (in case of MI and RFE), to obtain the key features with high diagnostic values. Then, the results were integrated and candidate biomarker genes across all techniques were identified.

### Twelve potential biomarker genes are identified by MI-SVM, RFE-SVM, and random forest models

Mutual information selection is used to obtain the best subset of features that can generate the highest accuracy score in differentiating between normal and LUAD/tumor samples. MI rank the genes in the dataset from the most to the least correlated to the two classes (normal and tumor). Utilizing the MI method, 45292 features (gene expression values) have been selected and ranked according to its importance. As a filtering technique, the MI produced an enormous number of features that did not minimize the feature space as expected.

**Fig 2:**
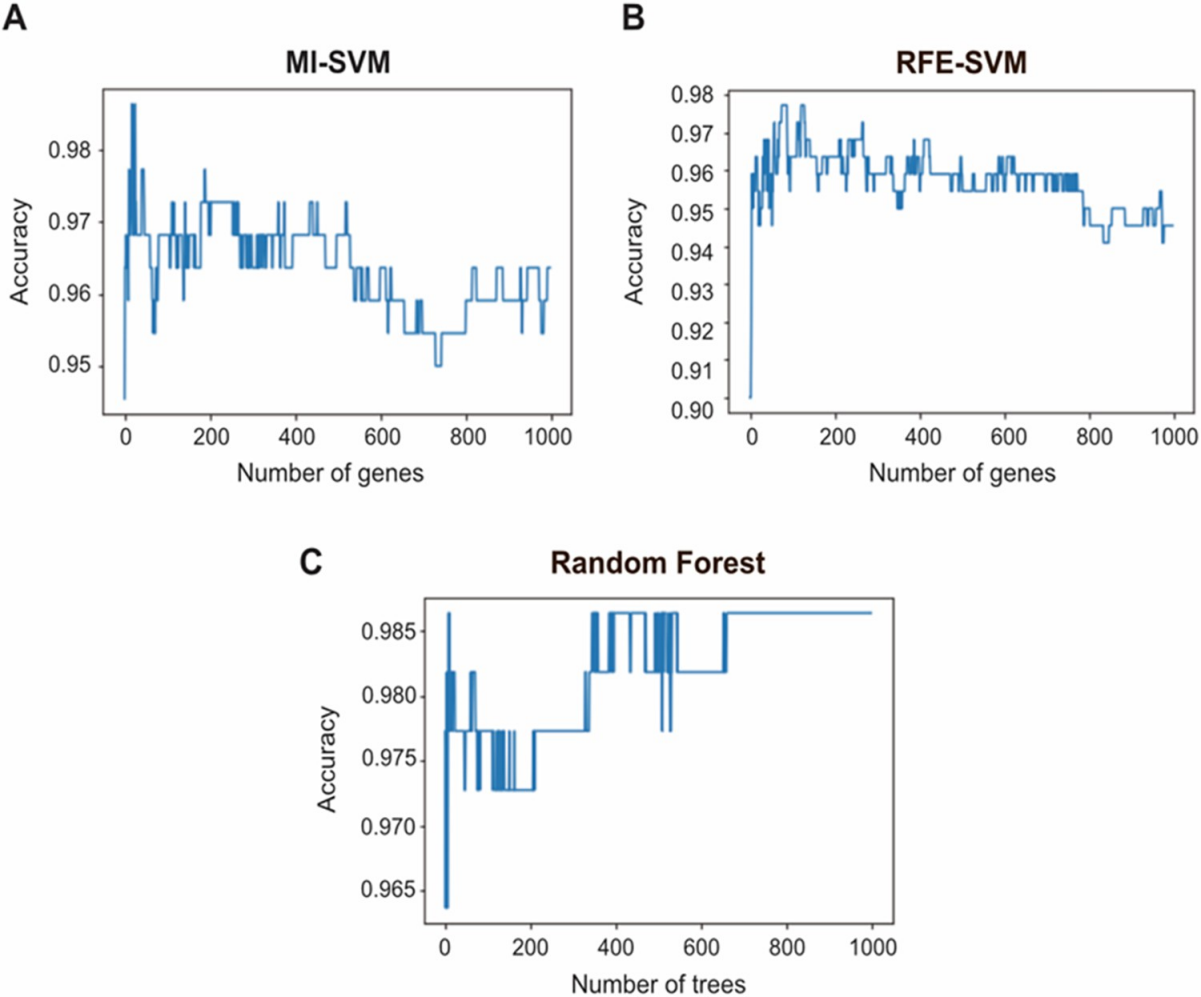
The incremental feature selection curves for the MI-SVM, RFE-SVM, and random forest models. The number of genes along with the corresponding SVM model weighted accuracy are shown (A and B) while the number of trees versus the RF achieved accuracy is shown in (C). (A) The peak of the curve is achieved at 19 genes with an accuracy of 98.64%. (B) The peak of the curve is achieved at 76 genes with an accuracy of 97.73%. (C) Utilizing 345 trees, the random forest model identified 1261 features and achieved an accuracy of 98.64%.

According to the ranked feature list, we followed a wrapping method utilizing SVM. We focused on the highest 1000 ranked features from the MI results. SVM was applied to consecutive feature subsets starting with the highly ranked two features. The first 19 MI-ranked features recorded the best weighted accuracy score of 98.64%. Fig 2A illustrates the accuracy achieved by the SVM classifier along with the different feature sets. The highest accuracy was achieved at 19 features, then a gradual decline happened with adding more features. The full list of the 19 MI-SVM features is listed in (S1 Table).

RFE is a wrapper technique in which data is split continuously until a desired subset of features is reached based on the chosen predictive model. We performed 1000 iterations to determine the best subset of features starting with one feature. The weighted accuracy score achieved with the least number of features was 97.73%, utilizing 76 features. Fig 2B illustrates the accuracy scores against the number of RFE-SVM features. The full list of the 76 candidate biomarkers is illustrated in (S2 Table).

**Fig 3:**
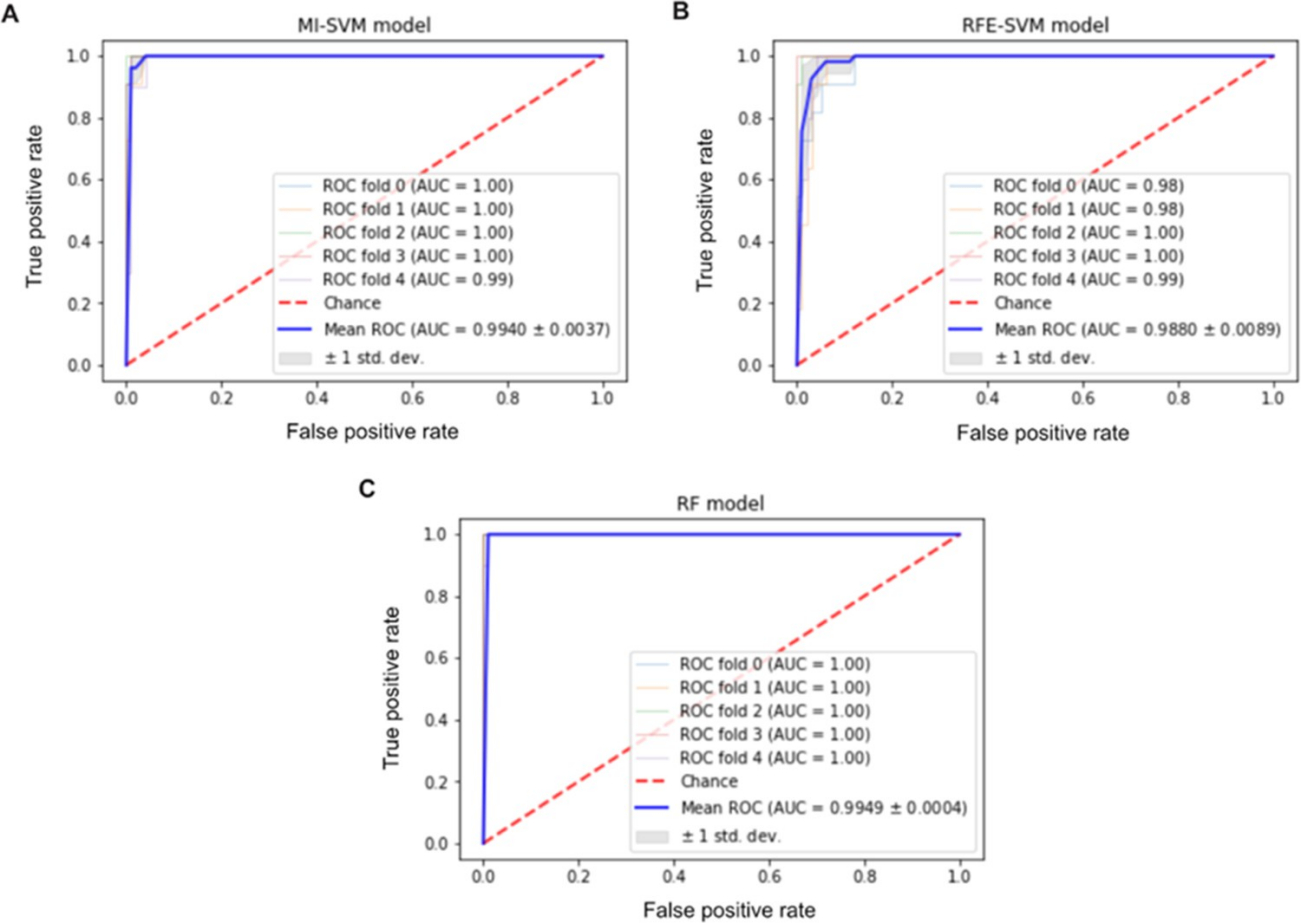
ROC and AUC analysis for different feature selection techniques. (A) MI-SVM model. (B) RFE-SVM model. (C) RF model.

Random forest is an embedded FS technique, where both feature selection and classification are performed together. In order to determine the best number of trees, we utilized different numbers of trees (up to1000 trees). Utilizing 345 trees, a performance of 98.64% was achieved. The resulting incremental feature selection curve is illustrated in Fig 2C. The random forest was generated using 1261 features, which are listed in (S3 Table). The different techniques used were compared in terms of precision, recall, specificity, balanced accuracy, and F1-score (Table 1). The Receiver Operating Characteristic (ROC) metric with stratified 5-fold cross-validation has also been calculated (Fig 3). The results are comparable, although the set of biomarker genes identified through each method is not quite identical. Most of the testing results of each feature selection method returned a high classification performance of over 93%. Specificity metric has ranged from 87% to 91%, indicating that the model had samples misclassified as LUAD. This can be due to the small number of the normal samples.

**Table 1:** A detailed evaluation table of MI-SVM, RFE-SVM, and RF models in terms of precision, recall, specificity, F1 score, and the mean AUC.

Technique	MI-SVM	RFE-SVM	RF
**Number of Features**	19 features	76 features	1261 features
**Precision**	0.9866	0.9778	0.9865
**Recall (Sensitivity)**	0.9864	0.9773	0.9864
**Specificity**	0.8773	0.9167	0.8773
**Balanced Accuracy**	0.9318	0.9470	0.9318
**F1-Score**	0.9859	0.9775	0.9859
**Mean AUC**	0.9940±0.0037	0.9880±0.0089	0.9949±0.0004

**Fig 4:**
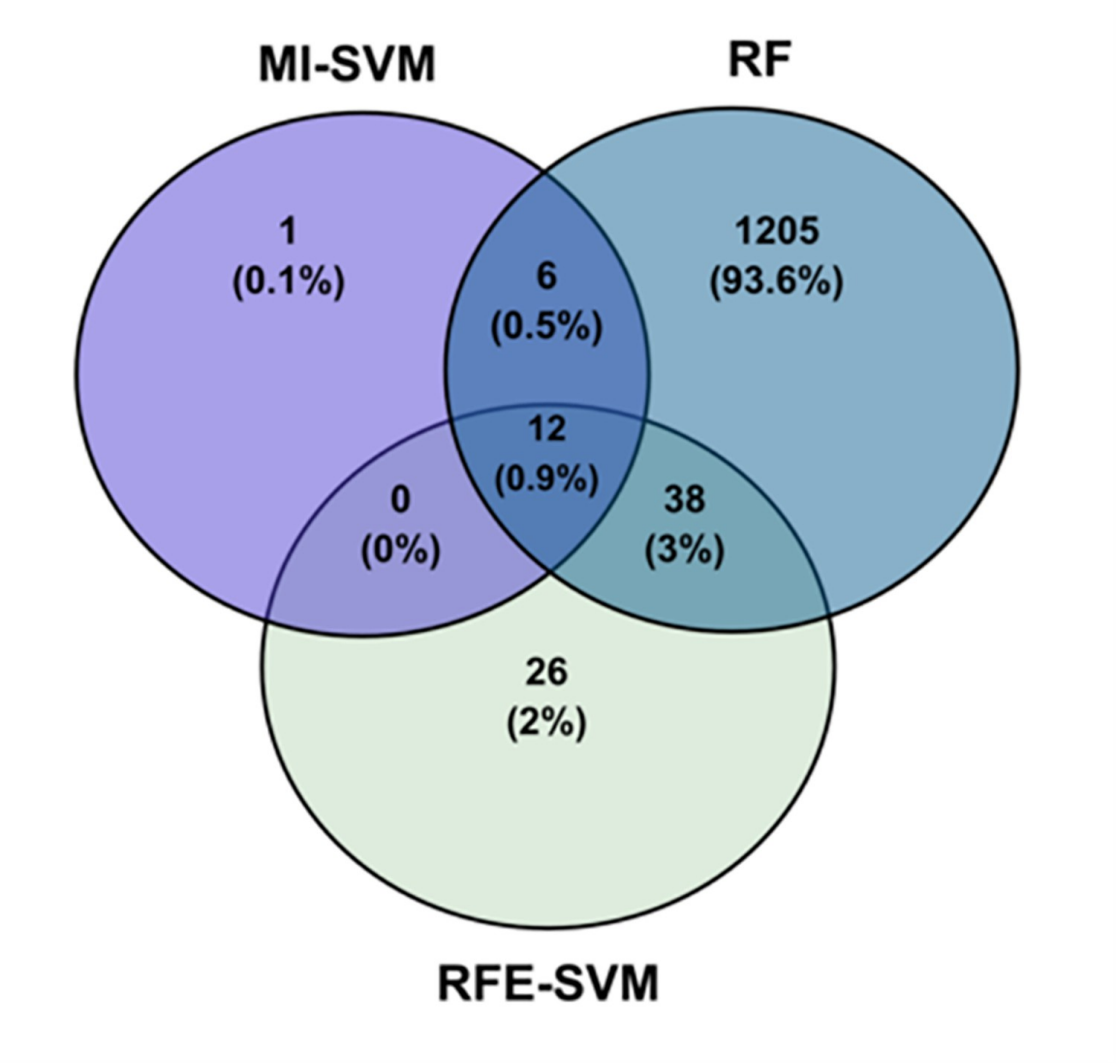
A Venn diagram illustrating the number of features of each model and the common features across all techniques.

The selected features reported by the MI-SVM, RFE-SVM, and RF were integrated as shown in (Fig 4). Overall, 12 features are reported as common between all methods. However, 44 features were additionally reported as common between at least two of the FS techniques. The MI-SVM and RF have 18 common features, which represent most of the features generated from the MI-SVM algorithm.

Regarding the 76 RFE-SVM features, 12 features are common with MI-SVM while 50 are common with RF features (Fig 4). As random forest has yielded the largest number of features, It was expected to have more features in common with other methods. Utilizing multiple well-known FS techniques maximizes the advantages of methods. The list of genes identified by all three methods or at least by two of the methods is presented in Table 2.

**Table 2:** List of features common between all selection techniques or common between at least two selection techniques.

Features common between all selection techniques	Features common between at least two selection techniques			
ADRB2	AC009093.3	CLDN18	LANCL1-AS1	SMAD6
AGER	AC025048.1	CLEC4M	LINC00656	SOX17
CAVIN2	AC104984.4	EPAS1	LINC00968	SPAAR
CLEC3B	ADGRE3	ERCC6L	NCAPGP2	SPOCK2
C10orf67	ADRB1	FCN3	NCKAP5	SSTR4
FABP4	ALAS2	FMO2	OTC	TEK
FAM107A/DRR1	ANGPT4	GPM6A	RGS9	TMEM100
LOC105376453	CAV1	GYPE	RTKN2	TNNC1
RGCC	CD300LG	HBA2	S1PR1	TOP2A
SFTPC	CD5L	HBB	SEMA3G	VIPR1
SLC6A4	CHRM1	HBM	SH3GL3	WNT3A
STX11				

**Fig 5:**
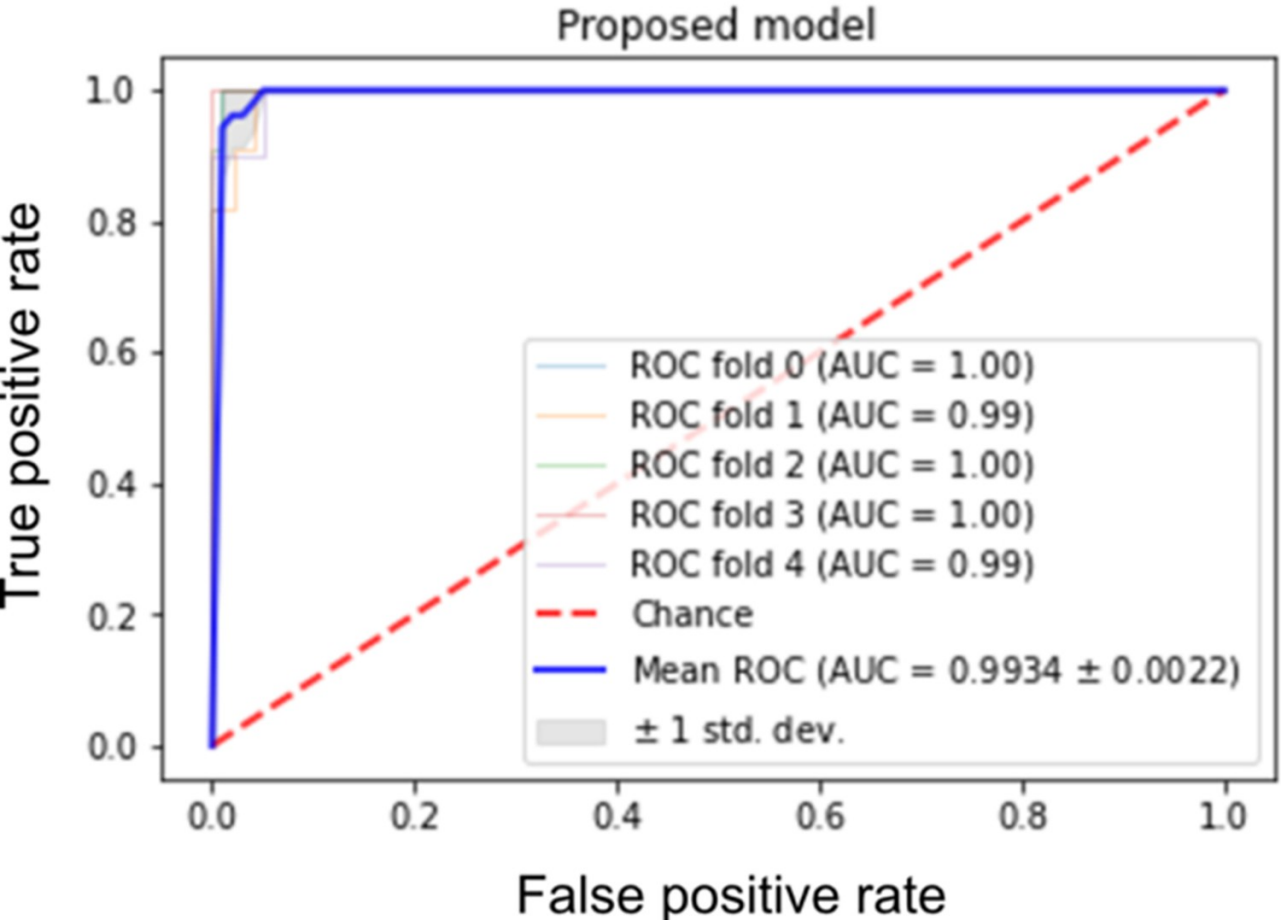
ROC and AUC analysis. Using the proposed model of the 12 candidate biomarkers with stratified 5-fold cross-validation.

**Fig 6:**
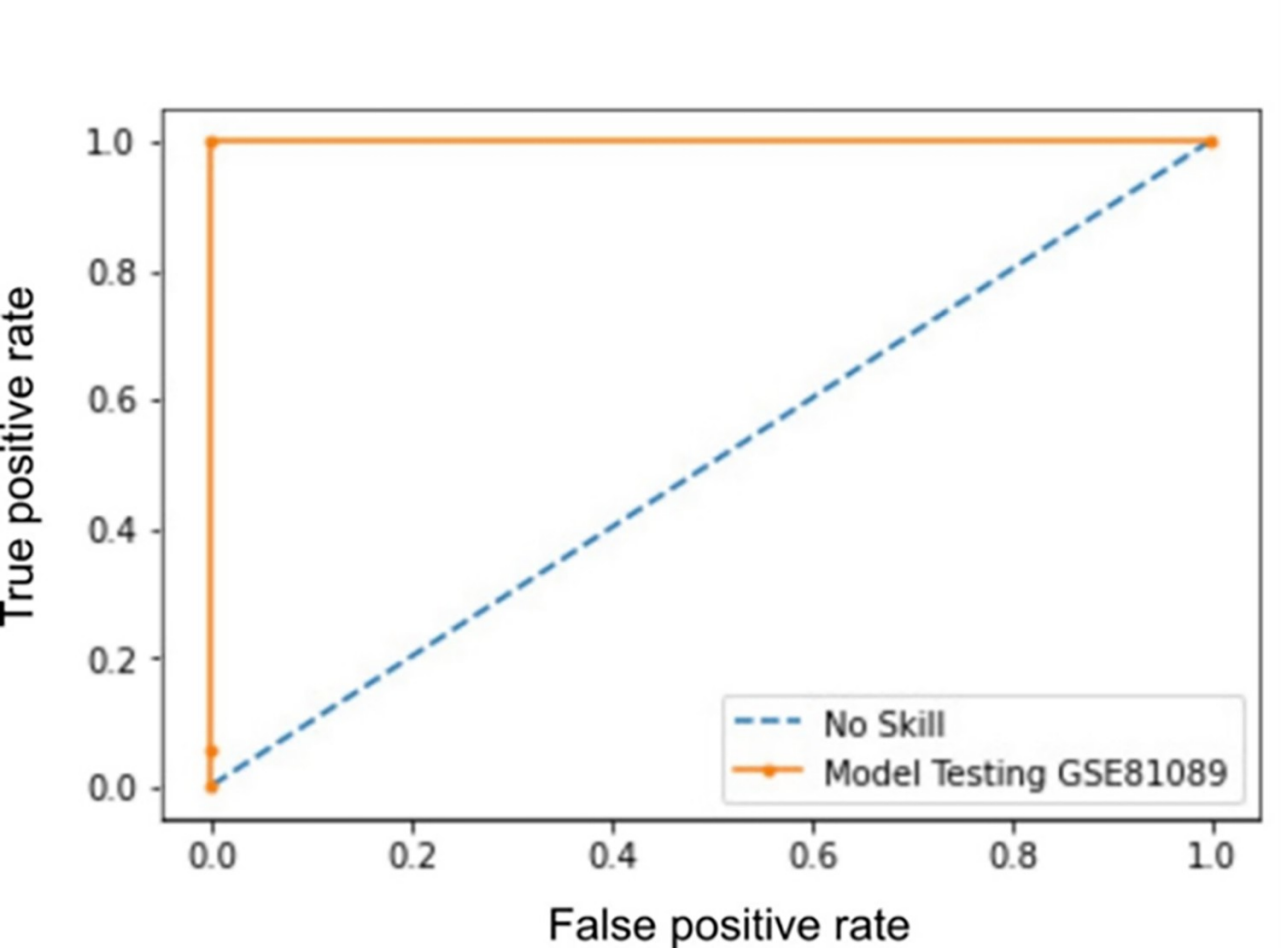
ROC and AUC validation of the proposed model using the external dataset (GSE81809).

To evaluate our candidate biomarkers reported by all techniques, an SVM model was constructed using only the 12 identified biomarker genes. The model achieved an accuracy score of 0.9799± (0.0069) using stratified 5-fold cross-validation. Other evaluation measures have also been computed (Table 3). The proposed model has achieved a mean AUC value of 0.9934±0.0022 with stratified 5-fold cross-validation (Fig 5). Furthermore, another SVM classification model was developed using the 56 features. This classifier achieved 97.27% accuracy. It is clear that utilizing only 12 genes yields comparable results with individual FS methods, but with a much smaller number of genes. Although Mutual information method performed well with relatively a small number of features, utilizing multiple methods reduces the number of candidate biomarkers with more biological relevance. An external dataset (GSE81809) was used to evaluate the proposed model (Table 3). Overall, all evaluation metrics indicate higher performance with over 92%. Fig 6 illustrates the ROC analysis with AUC value of 1.0000 using stratified 5-fold cross-validation.

**Table 3:** Evaluation statistics of the proposed model with the candidate biomarker using the testing samples and the external dataset.

	Precision	Recall (Sensitivity)	Specificity	Accuracy	Balanced Accuracy	F1-Score	AUC
**Proposed model (Testing)**	0.9768	0.9773	0.8359	0.9799± 0.0069	0.9066	0.9765	0.9934±0.0022
**Proposed model (External dataset)**	0.9649	0.9629	0.9259	0.9629	0.9444	0.9623	1.0000

**Fig 7:**
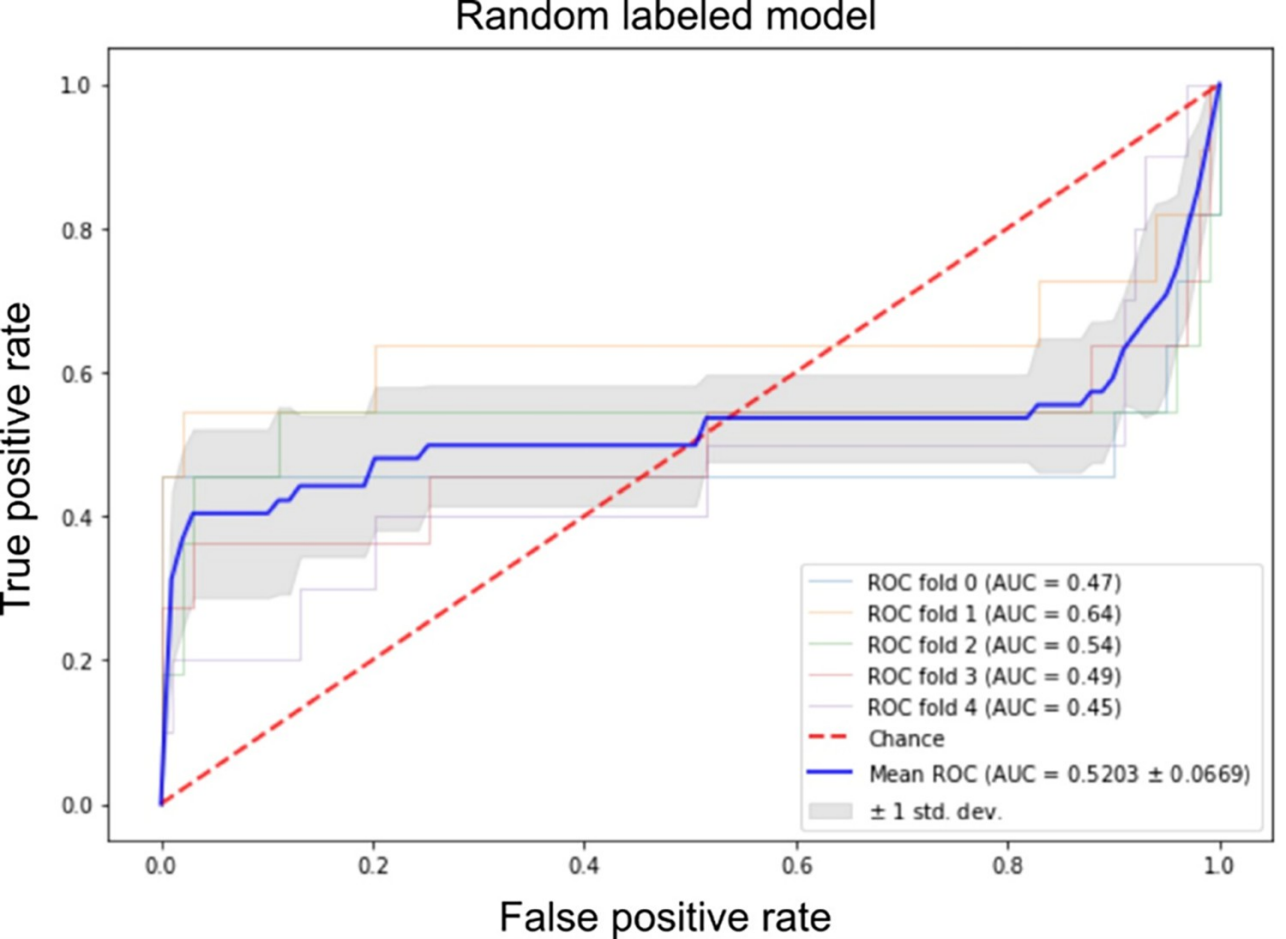
ROC and AUC analysis for a randomized version of the proposed model.

**Fig 8:**
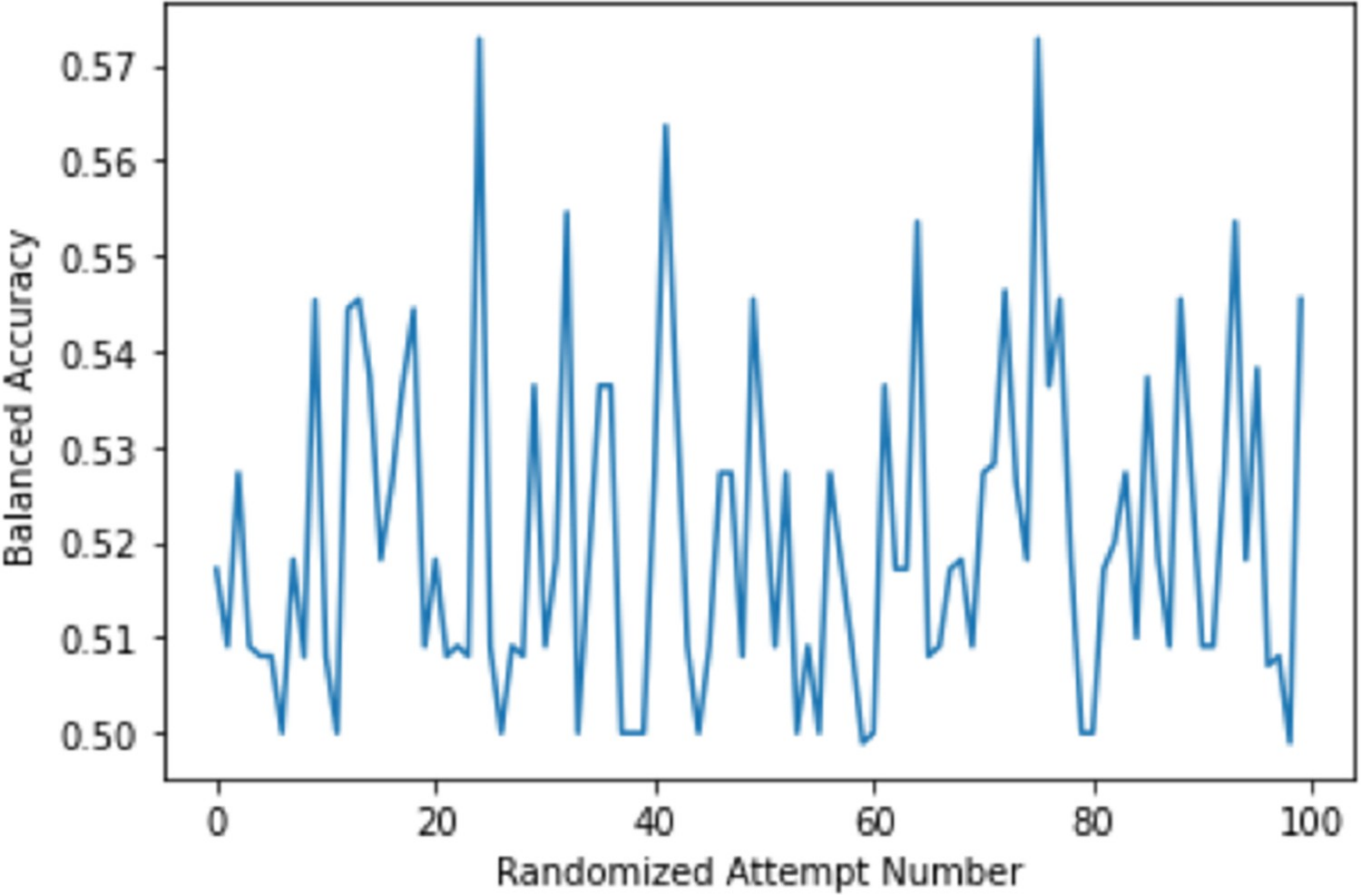
The balanced accuracy scores that were achieved by running 100 random labeled models. The X-axis is the attempt number. Y-axis indicates the balanced accuracy score.

To further support the output of our framework, we have developed a random labeled model where the training set labels have been randomized. Five-fold cross-validation was conducted where a balanced accuracy of 0.4990± 0.0020 was achieved. The mean AUC value of 0.5203± 0.0669 using 5-fold cross-validation has also been reported along with the ROC curves (Fig 7). Moreover, we have generated 100 random labeled models. The mean balanced accuracy of the generated models was 0.5208. Fig 8 is a summary figure to illustrate the balanced accuracy achieved by the random models.

### The candidate genes identified by the feature selection techniques are differentially expressed between normal and tumor samples

**Fig 9:**
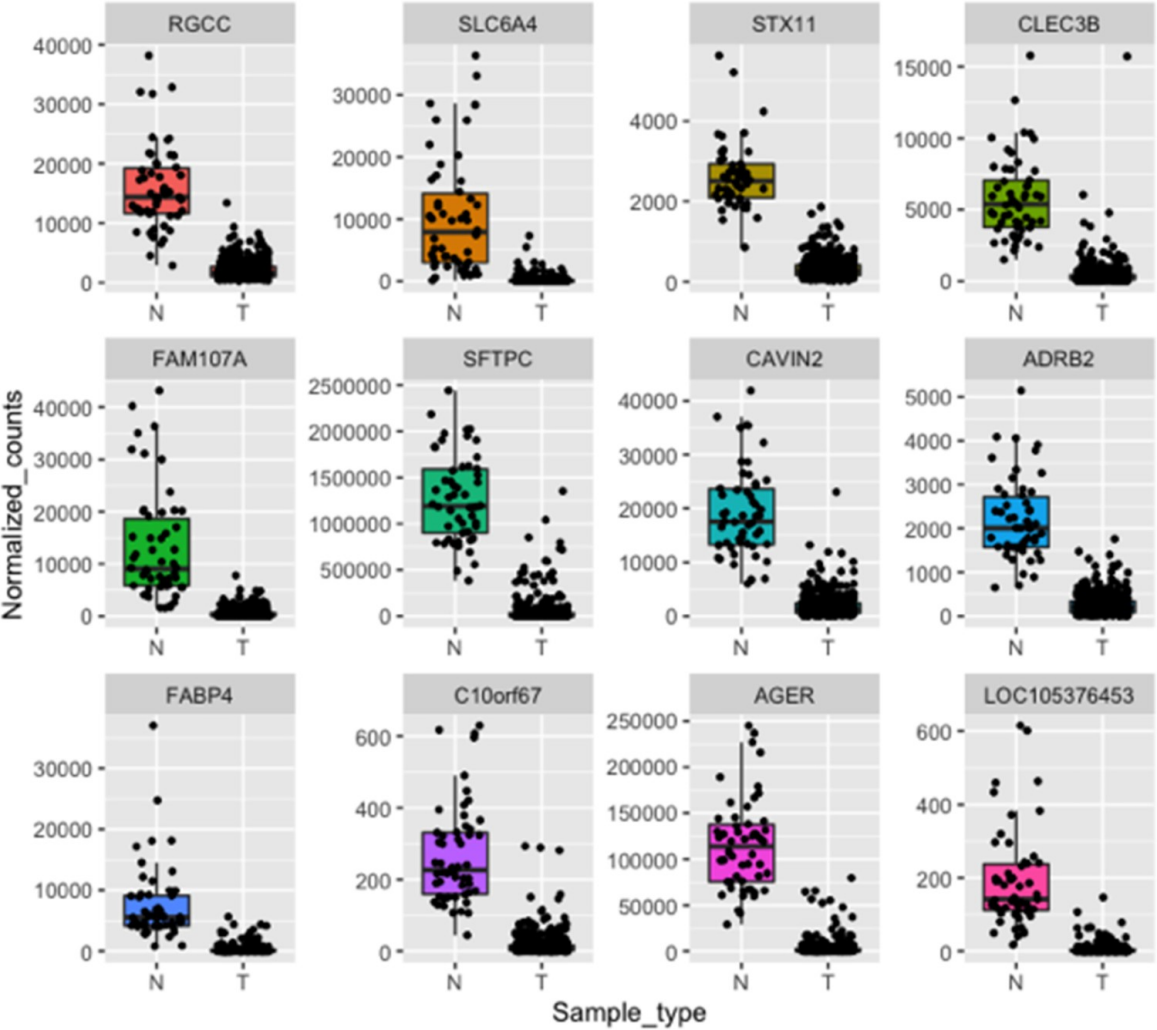
Boxplots representing the expression level of the 12 common candidate genes in LUAD patients in comparison to normal samples. N represents normal tissues and T represents tumor tissues.

**Fig 10:**
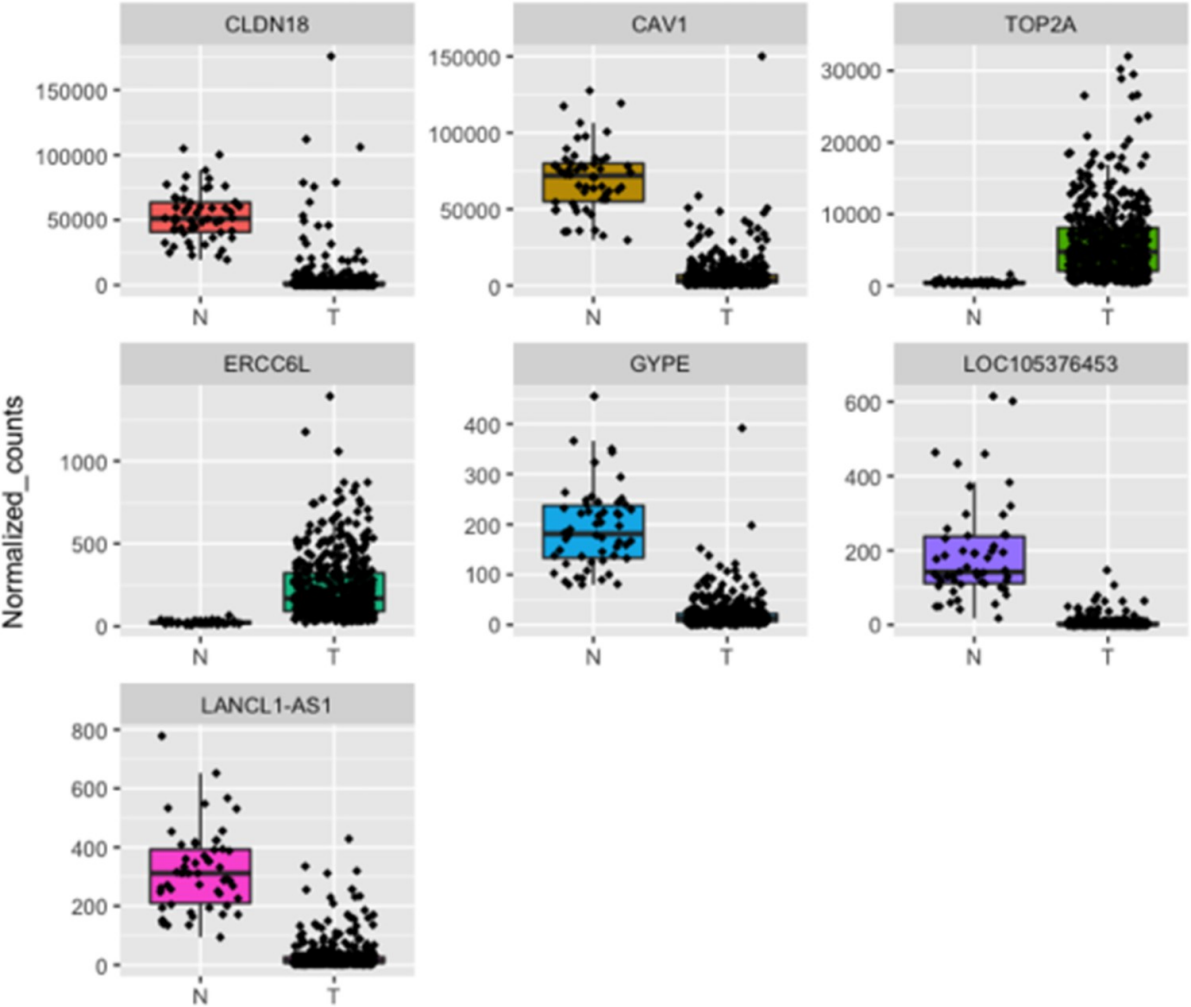
Boxplots representing the expression level of the MI-RF common genes in LUAD patients in comparison to normal samples. N represents normal tissues and T represents tumor tissues.

**Fig 11:**
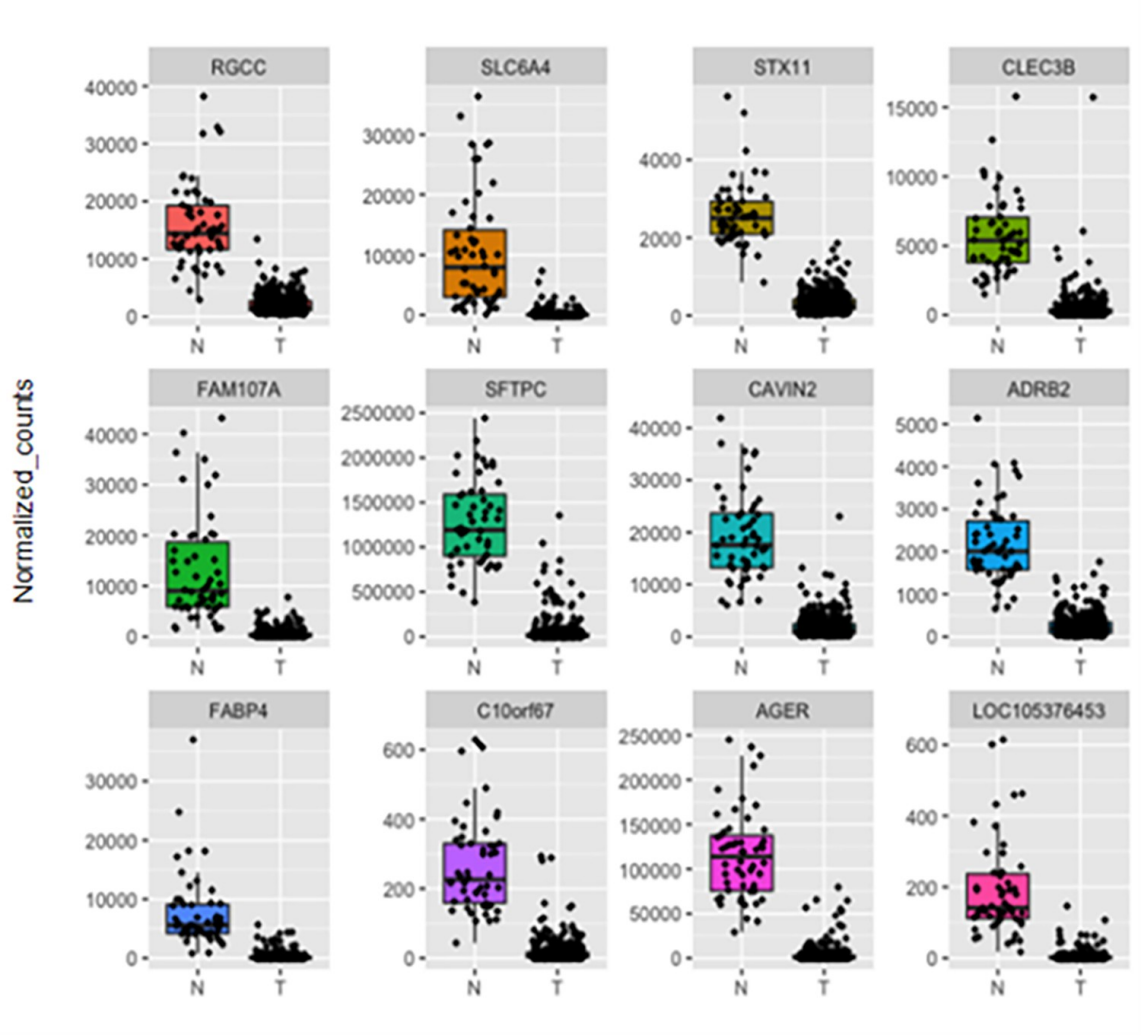
Boxplots representing the expression level of the RFE-MI common genes in LUAD patients in comparison to normal samples. N represents normal tissues and T represents tumor tissues.

**Fig 12:**
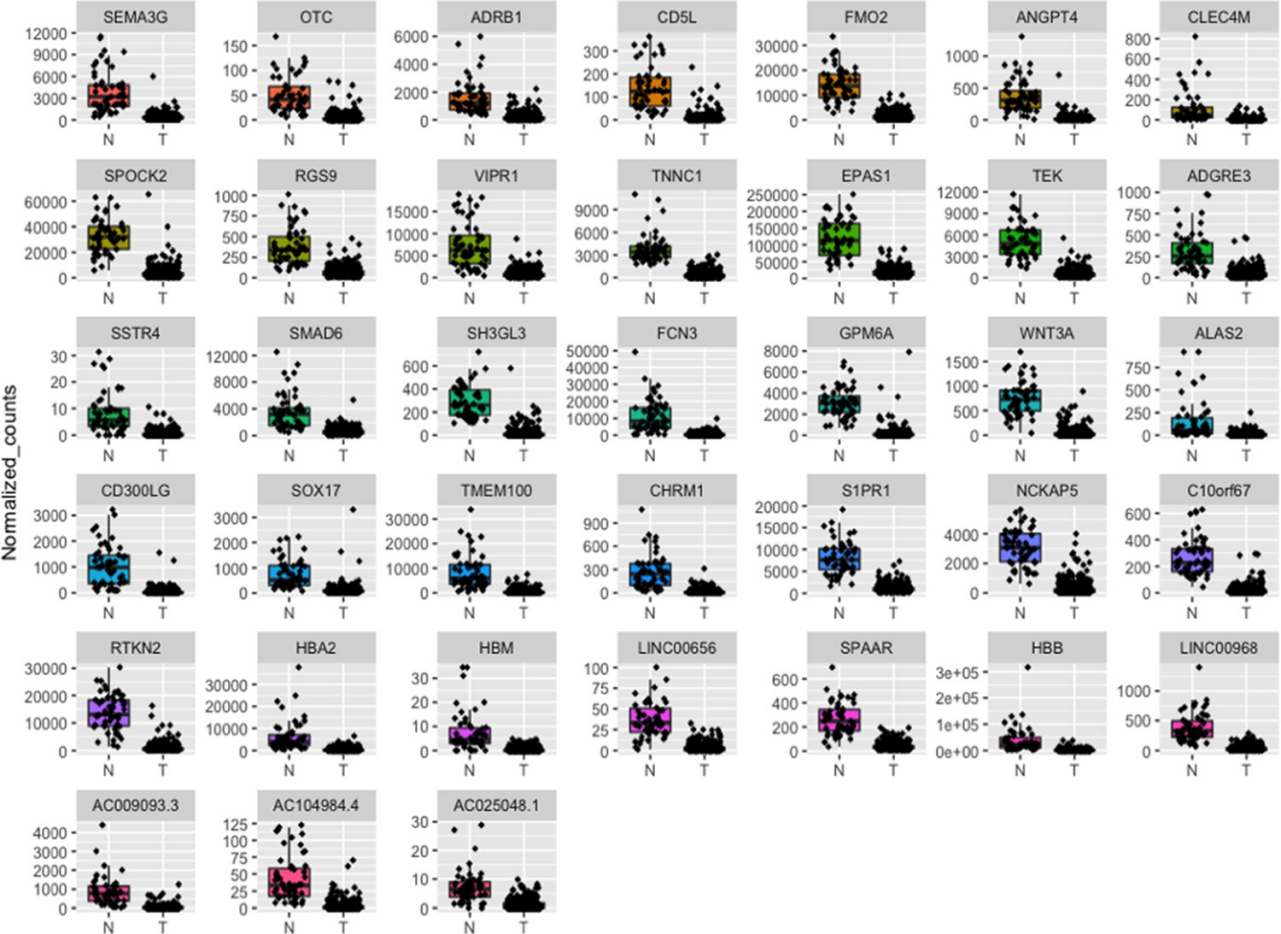
Boxplots representing the expression level of the RFE-RF common genes in LUAD patients in comparison to normal samples. N represents normal tissues and T represents tumor tissues.

To confirm the output of our framework, we performed differential expression analysis using DESeq2 [25]. We identified 5911 differential expressed genes (DEGs) between normal (N) and tumor (T) samples (S4 Table). Among the identified DEGs, we found that the 12 common genes obtained by the three different feature selection techniques are downregulated in tumor samples; this was also evident upon plotting the normalized counts of normal versus tumor samples (Fig 9). Similarly, upon plotting the normalized counts for the 44 genes identified by at least two selection techniques, we could find a trend where the majority of the genes are downregulated in tumors in comparison to normal samples. With the exception of TOP2A and ERCC6L, which were upregulated in tumor samples (S4 Table and Figs 10–12).

**Fig 13:**
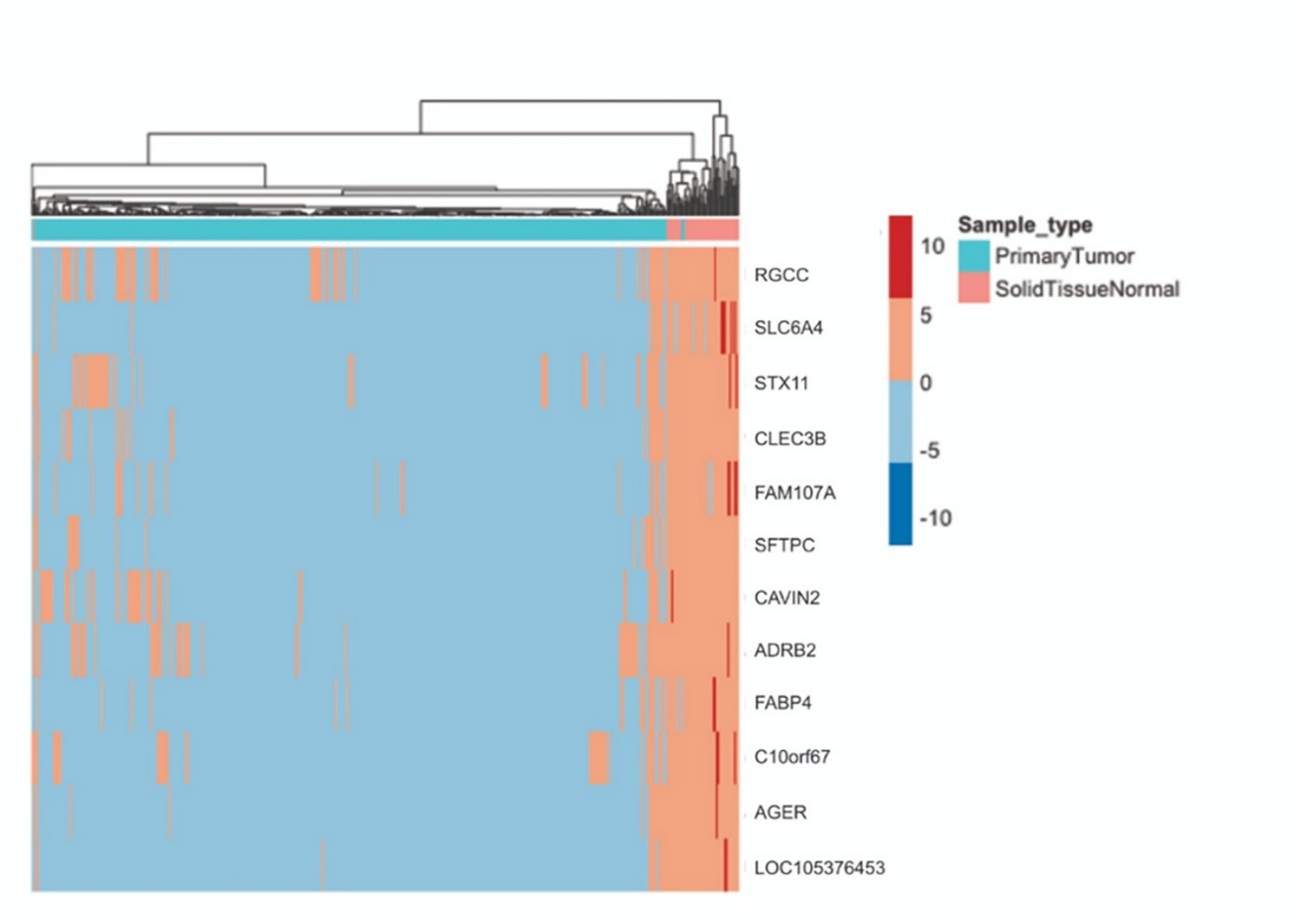
A heatmap representing the expression level of the 12 common candidate genes in LUAD patients in comparison to normal samples. Red represents up-regulation and blue represents down-regulation.

**Fig 14:**
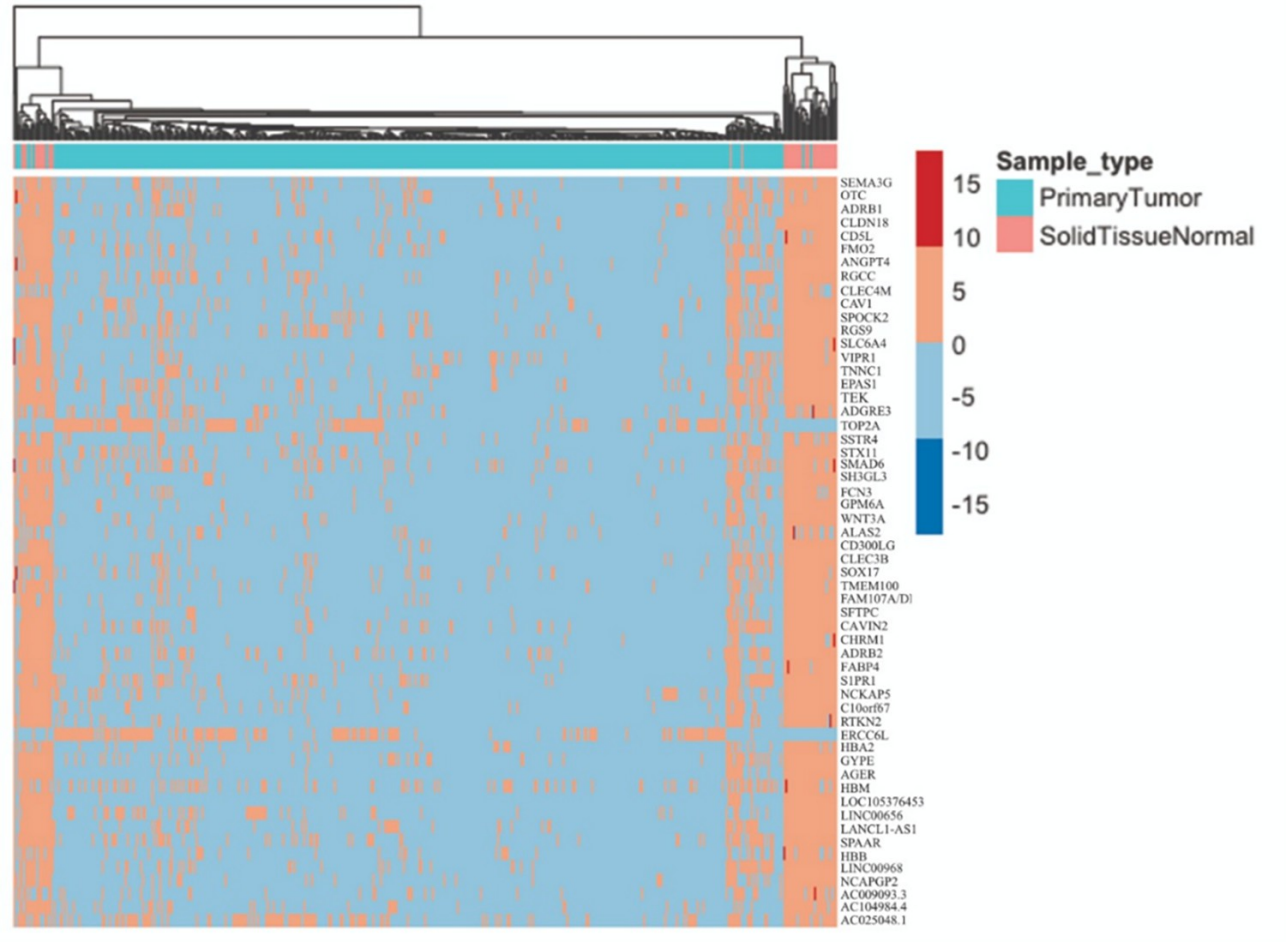
A heatmap representing the expression level of all genes obtained by at least two models. Red represents up-regulation and blue represents down-regulation.

Upon plotting heatmaps for the 12 common genes across all models (Fig 13) and the 44 genes common between at least two models (Fig 14), we could find that the tumor and normal samples appeared in separate clusters. This supports that our framework provides candidate genes that are highly correlated to LUAD and that can significantly differentiate between normal and tumor samples.

## Discussion

Previous studies have employed feature selection and machine learning methods for cancer diagnosis. For example, in [28], authors have used the same concept of overlapping feature selection techniques to identify biomarkers between lung adenocarcinoma and lung squamous cell carcinoma. Cai et al. [29] have focused on other significant types of lung cancer to identify DNA methylation markers utilizing ensemble-based feature selection techniques. Ma et al. [30] identified candidate biomarkers based on survival analysis data utilizing feature selection and classification. In [31], different types of data have been utilized, such as copy number variation (CNV) data, single nucleotide polymorphism (SNP), along with RNA-seq data. In this study, we applied a framework that combines feature selection methods and a prediction model to detect biomarker genes that differentiate between LUAD and normal samples. Our framework could identify 12 genes to be common between three different selection techniques. In addition, 44 genes were identified as common between at least two different selection techniques. We could further confirm the association of the 56 genes to LUAD via differential expression analysis. They were all identified as DEGs between LUAD and normal samples. Importantly, the vast majority of the 56 genes were previously correlated to LC in general or LUAD in different studies.

**Fig 15:**
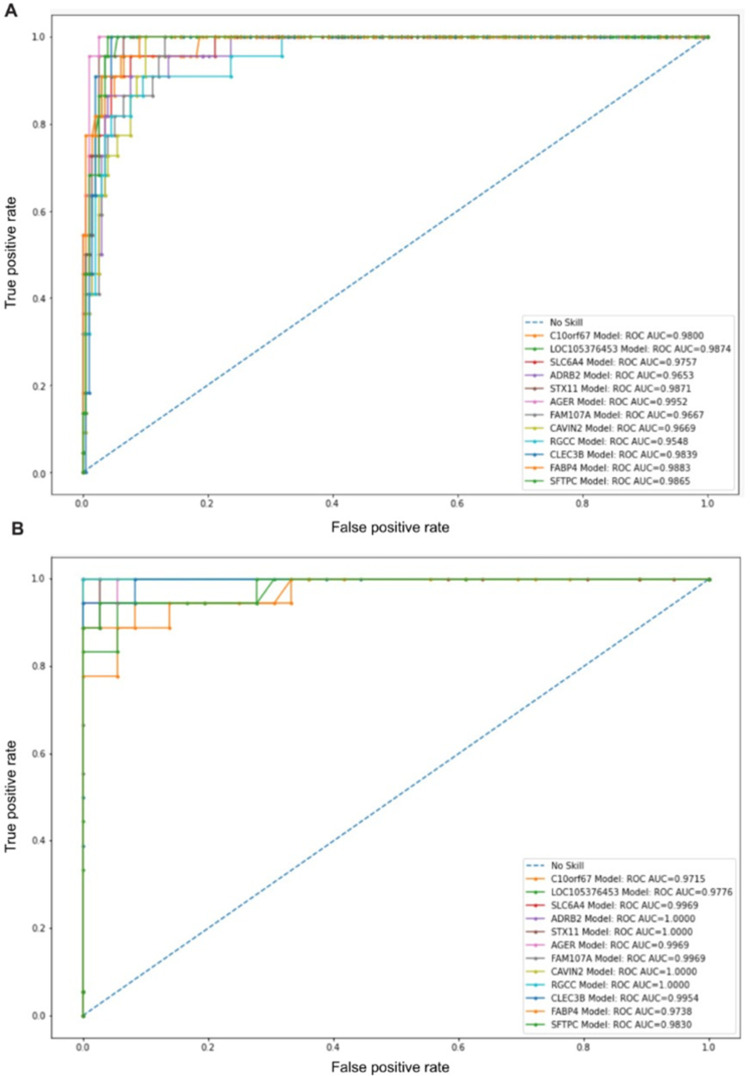
ROC curve analysis demonstrates the discriminating potential for the identified biomarkers. X-axis is the false positive rate (FPR). Y-axis indicates the true positive rate (TPR). Higher AUC suggests a higher discriminating potential for the gene. (A) The proposed model. (B) External dataset.

To evaluate the diagnostic potential of the twelve identified biomarkers, we have performed ROC curve analysis for each biomarker (Fig 15). All of the genes had areas under the curve (AUC) of over 0.95, with AGER being the highest, which suggests its most significant diagnostic potential in classifying LUAD. It has been shown that advanced glycosylation end-product specific receptor (AGER) is downregulated according to the subsequent downregulation of its regulator long non-coding RNA (lncAGER). Both AGER and lncAGER have an antitumor response; they cause apoptosis induction, inhibition of cell migration, invasion, and cell proliferation of the NSCLC cell line [32, 33]. Moreover, AGER has been reported to have a strong correlation with the tumor stage and overall survival rate of LUAD patients. Therefore, AGER is proposed to be a strong biomarker and prognostic agent for LUAD [33–35]. The high polymorphism of AGER is also considered a biomarker in the early diagnosis of LC. Furthermore, several genetic mutations in AGER are responsible for lung cancer development [36]. Polymorphism in ADRB2/β2-adrenergic receptor is also associated with lung cancer in the Chinese Han population [37].

We have repeated the same analysis using the external dataset GSE81089 for external validation to ensure that these results were reproducible. AUC and ROC were also used to analyze the 12 genes in the validation dataset (Fig 15). Primarily consistent with our results, all genes show AUC values well above 0.97.

In agreement with our results, we investigated the known association of our candidate genes with lung cancer. previous reports could show that FAM107A/DRR1 expression is significantly decreased in LUAD and non-small cell lung cancer (NSCLC) patients [34, 38]. CAVIN2 is also a tumor suppressor gene for NSCLC and its overexpression inhibits cancer proliferation. In addition, CAVIN2 expression increases the sensitivity of lung cancer cells to anticancer drugs [39]. SLC6A4 was also identified in a previous study as one of the most downregulated genes in LC [40]. CLEC3B is also downregulated in many lung cancer types (adenocarcinoma, squamous cell carcinoma, and large cell carcinoma) and its expression is correlated with the inhibition of LC proliferation. Therefore, it is suggested that it might act as a tumor suppressor gene for lung cancer. CLEC3B regulates immune infiltrating cells and since its regulation occurs at the early stages of lung cancer, it was suggested that it plays an important role in early prognosis [41]. RGCC was also shown to be downregulated in lung cancer patients according to the differential gene expression analysis of three different datasets; GSE18842, GSE19188, and GSE27262 [42]. STX11 and C10orf67 were downregulated in NSCLC patients as identified by bioinformatics analysis of several GEO datasets. In squamous cell lung carcinoma (SCC), ADRB2 was reported to be downregulated and its low levels were associated with lower survival [43]. Another study identified ADRB2 to be dysregulated in NSCLC [44].

In addition, SFTPC is one of the surfactant proteins in pneumocytes, which is essential for surfactant regulation in normal lung tissue. Its deletion was detected in NSCLC samples [45]. Other contrary to the other genes, FABP4 was reported to be highly expressed in NSCLC and it was associated with tumor node metastasis. It has been suggested as a lung cancer biomarker genes and its high expression is correlated with better NSCLC prognosis [46]. For LOC105376453, we did not find any reports about its association with lung cancer.

A large number of the 44 genes identified via at least two selection methods are also highly correlated with the prognosis or tumorigenesis of LC. For example, FMO2 was suggested as a tumor suppressor gene in LUAD [47]. SOX17 promoter is also highly methylated in NSCLC patients and it has a strong correlation with the overall survival rate of NSCLC patients [48]. TNNC1 was also validated as a tumor suppressor, which is downregulated in LUAD patients. Its low expression is strongly correlated with the invasiveness of LUAD cell lines and the increasing mortality rate among LUAD patients [49]. SPOCK2 was recently revealed to be a prognostic marker for LUAD. Low expression of SPOCK2 is correlated with a poor survival rate of LUAD patients [50]. According to differential expression analysis of microarray datasets, FCN3 was proposed to be a prognostic marker of LUAD due to its downregulation in tumors [51]. Moreover, VIPR1 was proposed as a prognostic marker for NSCLC. Its expression was downregulated in tumors, according to bioinformatics analysis and real-time PCR. In addition, it has been shown that VIPR1 expression is inhibited in metastatic LC [52]. TMEM100 was also reported to be downregulated in NSCLC and lung cancer cell lines. It acts as a tumor suppressor, where its knockout induces NSCLC proliferation and migration, and its activity promotes apoptosis in A549 and H460 cells through inhibiting the PI3K/AKT signaling pathway [53, 54]. CLDN18 was also proposed to be a tumor suppressor gene for LUAD. It regulates various oncogenic pathways and suppresses multiple malignant phenotypes *in vitro*. In addition, it inhibits tumor growth *in vivo* [55]. CAV1 is also highly correlated with the overall survival rate of LUAD. Its overexpression significantly suppresses the proliferation of LUAD cell lines; A549 and H157 [56]. GPM6A was suggested to be associated with apoptosis in small cell lung cancer (SCLC) [57]. SH3GL3 was also suggested to be a tumor suppressor of LC as its overexpression significantly suppresses cell proliferation and migration of LC cells. Additionally, SH3GL3 is negatively associated with the survival rate of LC patients [58].

In agreement with our results, TOP2A was reported to be overexpressed in LUAD, and its overexpression was correlated to LUAD progression. Results suggest that TOP2A act as a prognostic biomarker for LUAD as cell proliferation, migration, and invasion are significantly inhibited in A549 and GLC82 cells lacking TOP2A [59]. In disagreement with our findings, RTKN2 gene was shown to be upregulated in NSCLC. Its knockout inhibits cell proliferation of NSCLC cells and colony formation [60]. S1PR1 upregulation is also involved in various tumorigenesis processes, cell proliferation, and invasion [61, 62]. Furthermore, EPAS1 is overexpressed in PC14HM NSCLC cell line and by knocking it out, the proliferation of PC14HM cells and the tumorigenesis were inhibited [63]. SMAD6 was reported to support the growth and survival of lung cancer. Therefore, it was suggested to be a target for inactivation as a new treatment approach [64]. The remaining genes; CD5L, WNT3A, CLEC4M, RGS9, SEMA3G, ERCC6L were also highly correlated to tumors, but little or no evidence for association with lung cancer is reported [65–70]. We believe further investigation can reveal strong connections, as interestingly the association of SH3GL3, TNNC1, SPOCK2, VIPR1, and RTKN2 with LC was reported in very recent years [49, 50, 52, 58, 60].

In summary, we believe the combination of the three feature selection techniques provides more reliable outcomes and could help in identifying novel biomarkers. Consequently, improving the current diagnostic approaches and enabling better tailoring for precision medicine. All 12 genes have a strong correlation with LC as well as a large number of the 44 genes. All our candidate genes were downregulated in this study, with the exception of TOP2A and ERCC6L. However, other studies reported the overexpression of some of the genes. This could be owed to biological differences between the patients’ samples analyzed in this study and previous studies [71]. Analysis of cell lines versus a cohort of patients can also result in output variability [72].

Overall, the consistency between the output of our framework, differential expression analysis, and previous reports gives confidence in our approach and supports the usage of the three different feature selection techniques together to identify biomarkers, instead of relying on a single selection method.

## Methods

### Data retrieval

LUAD RNA-seq data used in this study was obtained from The Cancer Genome Atlas (TCGA). To eliminate any bias or distortion in the data, we only used normal and primary tumor samples; no recurrent tumor samples. Moreover, only tumor samples with disease type “adenomas and adenocarcinomas” were used. We used the raw transcriptome profiling data (HTSeq–Counts). The used phenotypes were divided into two classes, which were “Solid Tissue Normal” and “Primary Tumor”. The number of the normal samples and tumor samples were 54 and 495, respectively with a total number of 549 samples. A total of 60,488 genes have been included and analyzed.

We also retrieved another dataset of primary non-small cell lung cancer and their normal tissues from GEO for external validation. Raw counts were retrieved under the accession number of GSE81089 [73] selecting only LUAD samples. The LUAD dataset consists of 54 samples; 36 tumor samples extracted from tumor and 19 normal samples. A representation for the data cohort has been shown in Table 4.

**Table 4:** A graphical overview of the data cohort.

Cancer Type	Source	Description	Tumor samples	Normal samples	Total samples
**LUAD**	TCGA	Used for model establishment (Standard training and testing)	495	54	549
**LUAD**	GEO (GSE81089)	Used for model external validation (selecting only LUAD samples)	36	19	54

### Data preprocessing

Features were normalized to have zero mean and unit variance as follows: $z=\frac{x-u}{s}$, where z is the normalized expression value, x is the expression value of each gene, u is the mean of the expression values across the gene samples, and s is its standard deviation [74–76]. Data was split into 60% (329 samples) for training and 40% (220 samples) for testing.

### Mutual information

Mutual information (MI) algorithm measures the relevance of the features to the classes and the redundancy of the features with each other. It can measure the association of a random feature based on another. In case of genes, a higher mutual information value amongst two genes means that those two genes are associated with each other in a non-random manner. MI was modified to avoid the binning problem by using a k-neighbors estimator. The MI K-neighbors method can be used to detect discrete classes (cancerous or non-cancerous) based on continuous values (expression levels) [16].

The MI k-neighbors method was applied to our data with k = 3 and features were sorted according to its MI feature importance values. Many features had very low or zero MI values. Features with zero values were eliminated. The top 1000 features were evaluated iteratively with SVM to decide the best subset of features.

### Support vector machine

SVM is a supervised learning technique and is considered to be one of the powerful tools for classification [77]. It identifies the decision boundary between the data as a hyperplane which is designed to be as far as possible to the closest samples of each class; those samples are known as support vectors [78]. For any dataset, where $\left(x_{1},y_{1})\to (x_{n},y_{n}),x_{i}\in R^{d}\;and\;y_{i}\in (-1,+1\right)$. X represents the feature set and Y represents the class labels. To obtain the maximum margin hyperplane through training an SVM model, we seek to solve the following convex quadratic programming problem [79]:

$$L(\alpha )=\sum_{i=1}^{n}\alpha_{i}-\frac{1}{2}\sum_{i.j}^{n}\alpha_{i}\alpha_{j}y_{i}y_{k}K(x_{i}{,x}_{j})$$

Under the constraints

$$\sum_{k=1}^{n}\alpha_{k}y_{k}=0,\alpha_{k}\geq 0$$

Where *n is the number of data points, α′s* are the Lagrange multipliers, and *K* is the kernel function. We applied SVM with a linear kernel with features selected using MI and RFE methods for our framework. All other parameters have been set to default.

### Recursive feature elimination

Recursive feature elimination (RFE) is one of the commonly used wrapper-based FS techniques. RFE is a greedy algorithm to find the best subset of features giving the highest performance. It generates a subset of the features while keeping the best subset at each iteration. A rank of all features based on their elimination order is then obtained. The elimination criterion is based on the chosen predictive model. SVM with linear kernel have been used here as the predictive model. The methodology of SVM-RFE was proven to be very efficient in feature selection to eliminate redundant genes [17].

In our framework, a step has been set to 0.5. That means 50% of the features were eliminated at each iteration. The algorithm splits the training data into two equal parts at each iteration and keeps the part that outputs the higher score based on the SVM estimator. RFE has been employed iteratively with the number of features ranges from (1–1000) to determine the best set of features that achieves the highest accuracy. Other parameters have been set to default.

### Random forest model

Unlike filter and wrapper-based techniques, embedded method selects significant features as part of constructing the model. Random forest is constructed utilizing multiple decision trees for prediction [80]. Classification and Regression Trees (CART) is utilized along with the bagging technique [81]. As RF is built with many decision trees, each tree outputs a specific prediction. The majority vote of the resulted predictions is taken into consideration. We have utilized RF with a different number of decision trees (up t0 1000 trees) to identify the optimal subset of features. Other parameters have been set to default.

### Differential expression analysis and candidate genes visualization

Differential expression analysis was performed via DESeq2. Only solid tissue normal and LUAD primary tumor samples were selected. The adjusted p-value (padj) and log fold change (LFC) were utilized to detect the statistically significant DEGs with a threshold padj < 0.05 and LFC > 2. The normalized counts were implemented by estimateSizeFactors of DESeq2. Visualization of boxplots and heatmaps were implemented using ggplots [82] and pheatmap (https://cran.r-project.org/web/packages/pheatmap/index.html) packages in R.

## Conclusion

Identifying gene expression signature that differentiates between tumor and normal samples from differential expression analysis of RNA-seq data is a major challenge. The analysis reveals a huge number of genes and thus, extracting the disease-associated genes from such data accurately is a difficult task. Utilizing an ensemble of FS techniques has proven its robustness and reliability in identifying accurate and biologically relevant biomarker genes. In our framework, we utilized mutual information and recursive feature elimination methods along with the SVM classifier model. We have also utilized random forest as an embedded FS technique. Our framework has identified 12 candidate biomarkers across all methods where a previous association with LC has been shown. The differential expression analysis also confirmed their dysregulation in LUAD. We propose that our framework can be applied to different types of cancers and other complex diseases to enable the identification of novel biomarkers. This is especially important for developing countries, where narrowing down the candidate genes for personalized assessment is needed to diagnose patients in a cost-effective manner. Such an approach also fits well to population data, where identifying the most correlated genes in a specific population and investigating them further on an individual patient level would greatly improve diagnosis and decrease disease burden.

## Supporting information

S1 Table The candidate gene list identified by MI-SVM model. (XLSX)

S2 Table The candidate gene list identified by RFE-SVM model. (XLSX)

S3 Table The candidate gene list identified by random forest model. (XLSX)

S4 Table The List of identified differential expressed genes (DEGs) between normal (N) and tumor (T) samples. (XLSX)
